# High Risk of Hepatitis B Reactivation among Patients with Acute Myeloid Leukemia

**DOI:** 10.1371/journal.pone.0126037

**Published:** 2015-05-14

**Authors:** Chien-Yuan Chen, Sheng-Yi Huang, Aristine Cheng, Wen-Chien Chou, Ming Yao, Jih-Luh Tang, Woei Tsay, Wang-Huei Sheng, Hwei-Fang Tien

**Affiliations:** 1 Division of Hematology, Department of Internal Medicine, National Taiwan University Hospital, Taipei, Taiwan; 2 Division of Infectious disease, Department of Internal Medicine, National Taiwan University Hospital, Taipei, Taiwan; 3 Department of Laboratory Medicine, National Taiwan University Hospital, Taipei, Taiwan; 4 Tai- Cheng stem cell therapy center, National Taiwan University Hospital, Taipei, Taiwan; Taipei Veterans General Hosptial, TAIWAN

## Abstract

**Background:**

Hepatitis B virus (HBV) infections are common and associated with significant morbidity and mortality in cancer patients. However, the incidence and risk factors of HBV reactivation in patients with acute myeloid leukemia (AML) are rarely investigated.

**Methods:**

AML patients followed-up at the National Taiwan University Hospital between 2006 and 2012 were analyzed. The clinical characteristics and laboratory data were retrospectively reviewed.

**Results:**

Four hundred and ninety patients comprising 265 men and 225 women were studied. The median age was 52 years (range, 18 - 94). Chronic HBV carriage was documented at the time of leukemia diagnosis in 57 (11.6%) patients. Forty-six (80.7%) of the 57 HBV carriers received prophylaxis with anti-HBV agents. Sixteen HBV carriers (28.1%) developed hepatitis B reactivation during or after chemotherapy, including 7 patients who had discontinued antiviral therapy. The incidence of hepatitis B reactivation among AML patients with HBV carriage was 9.5 per 100 person-years. Prophylaxis with anti-HBV agents significantly decreased the risk of hepatitis B reactivation among HBV carriers (13% vs. 61%, p<0.001). Four (2.8%) of 142 patients with initial positive anti-HBsAb and anti-HBcAb experienced hepatitis B reactivation and lost their protective anti-HBsAb. Multivariate analysis revealed that diabetes mellitus (p=0.008, odds ratio (OR) = 2.841, 95% confident interval (CI): 0.985-8.193) and carriage of HBsAg (p<0.001, OR=36.878, 95% CI: 11.770-115.547) were independent risk factors for hepatitis B reactivation in AML patients.

**Conclusions:**

Hepatitis B reactivation is not uncommon in the HBsAg positive AML patients. Prophylaxis with anti-HBV agent significantly decreased the risk of hepatitis B reactivation.

## Introduction

Acute myeloid leukemia (AML) is a heterogeneous disorder with regards to the morphology and chromosome aberrations detected in the leukemic cells [1]. In spite of this, most AML patients receive standard induction chemotherapy containing anthracycline and cytarabine, and consolidation with high dose cytarabine [1]. The chemotherapy agents used for AML are similar to those used for lymphoma [2–4]. High dose cytarabine infusion can cause skin rash and fever [5], and thus, steroid is usually added as prophylaxis. Abnormal liver function tests are frequently found among AML patients receiving chemotherapy, possibly related to the chemotherapy itself, the concomitant use of antifungal agents and total parenteral nutrition, or to sepsis related organ dysfunction, leukemic infiltration, or graft versus host disease. Reactivation of viral hepatitis, including hepatitis B, is also a potential cause for abnormal liver function tests in leukemic patients receiving chemotherapy [6, 7].

Hepatitis B virus (HBV) affects 350 to 400 million persons worldwide and constitutes a major global health burden [8]. HBV is a DNA virus transmitted parenterally, sexually, and perinatally [8]. HBV infection can cause acute and chronic liver disease including cirrhosis and hepatocellular carcinoma. Following immunosuppression, HBV replication along with signs of hepatocellular injury in a silent hepatitis B surface antigen (HBsAg) carrier may occur [9]. The clinical presentation of hepatitis B reactivation is variable, ranging from asymptomatic to fulminant hepatitis, liver failure, and death. Previous reports of HBV reactivation have been observed in patients with lymphoma [10, 11], treated with corticosteroids [12, 13] and rituximab [14, 15] as well as in patients undergoing stem cell and bone marrow transplantation [16, 17]. However, the epidemiology and clinical manifestations of hepatitis B reactivation among AML patients are rarely described [10]. Taiwan has long been an area endemic for HBV, and, previously, the seroprevalences of anti-HBc approached 80–90% and of HBsAg 15–20% prior to the nationwide hepatitis B vaccination program [18]. In this study, we retrospectively reviewed the epidemiology, clinical and laboratory data of hepatitis B in AML patients at a medical center to understand the epidemiology and clinical outcomes of HBV reactivation among AML patients.

## Patients and Method

### Ethics Statement

The Institutional Review Board of the National Taiwan University Hospital Research Ethics Committee waived the need for written informed consent from the participants in the retrospective review of medical record and approved this study. This research conformed to the Helsinki Declaration and local legislation, and was approved by the Institutional Review Board National Taiwan University Hospital Research Ethics Committee.

### Hospital setting and patients

National Taiwan University Hospital (NTUH) is a 2900-bed teaching hospital in northern Taiwan providing both primary and tertiary care. In this study, the clinical and laboratory data, hepatitis B serology, hepatitis B virus DNA and outcome of all adult AML patients during the period January 2006 to December 2012 at NTUH were analyzed retrospectively by chart review. Liver function tests, hepatitis B serology titers, and HBV DNA levels were performed as clinically indicated.

### Definitions

The serum biochemistry (alanine transaminase, ALT) was measured by the Beckman Coulter AU5800 platform (Beckman Coulter Inc, Brea, CA, USA); HBsAg, anti-HBsAb, Anti-HBc, HBeAg, and anti-HCV were performed with Abbott ARCHITECT i2000SR (Abbott Laboratories, Abbott Park, North Chicago, IL, USA); HBV DNA was analyzed with COBAS AmpliPrep /COBAS TaqMan HBV Test, v2.0 (Roche, Basel, Switzerland) according to the manufacturer’s instruction. The serological results were defined by the following cut-off values: positive hepatitis B surface antigen (HBsAg) if≧ 0.05 IU/mL; positive anti-hepatitis B surface antibody (anti-HBsAb) with a threshold of 10 mIU / mL [19]; positive anti-hepatitis B core antibody (anti-HBcAb) if ≧ 1.0. Chronic hepatitis B carrier status was defined by the detection of positive hepatitis surface antigen (HBsAg) for more than 6 months. Hepatitis B reactivation was defined as a greater than 10-fold increase, compared with previous nadir levels, of HBV DNA or by the reappearance of hepatitis B “e” antigen in the serum for patients whose baseline HBeAg was negative. HBV-related hepatitis was defined as a greater than 3-fold increase of ALT level (the upper normal limit is 41 U/L at NTUH) accompanying or following HBV reactivation. In July 1984, the Taiwan government launched a nationwide universal HBV vaccination program [20]. The following catch-up program of the nationwide HBV vaccination had a coverage rate of 86.9 to 98.0% [21].

The diagnosis of cirrhosis was based on clinical symptoms / signs and imaging study (ultrasound, computed tomography). The diagnostic criteria of hepatocellular carcinoma(HCC) in this study was based on histologic and/or clinical findings and on the presence of all of the following criteria: chronic viral hepatitis infection and liver cirrhosis, hepatic tumor with imaging (ultrasound, computed tomography) characteristics compatible with a diagnosis of HCC and without evidence of gastrointestinal or other primary tumor, and a persistent elevation of the serum level of alpha-fetoprotein (AFP) to 400 ng / mL or higher.

### Chemotherapy of acute myeloid leukemia

Standard induction chemotherapy consisting of cytarabine and anthracycline were used for patients with AML at NTUH. Standard dose chemotherapy was defined as cytarabine 100mg / m^2^ body surface area administered as a continuous infusion for seven days. Low dose chemotherapy was defined as cytarabine 10 to 20 mg / m^2^ body surface area for 7 to 14 days. Consolidation chemotherapy consisted of high-dose cytarabine-based regimens. Steroid was used routinely for prevention of fever and skin rash in AML patients receiving high dose cytarabine chemotherapy. Methylprednisolone 40 mg twice daily by intravenous injection was administered before cytarabine for 6 to 8 doses. Patients with acute promyelocytic leukemia (APL) were treated with all trans retinoic acid (ATRA) in combination with anthracycline-based chemotherapy. Hypomethylating agents such as azacitidine were given at a dose of 75 mg / m^2^ body surface area for 7 days every 4 weeks.

### Statistical analysis

Survival was estimated by the Kaplan-Meier analysis and compared using the log-rank test. Categorical variables were compared using the Chi-square test. Univariate and multivariate analyses of factors associated with time to HBV reactivation were performed using the Cox proportional hazards model. Factors with p-value of ≤0.10 in the univariate analysis were considered in the multivariate model. All statistical analyses were performed using the statistical package SPSS for Windows v.18 (SPSS Inc., Chicago, IL). A p-value of ≤0.05 was considered significant and all statistical tests were two-tailed.

## Results

### Epidemiology

There were 593 AML patients diagnosed and regularly followed at NTUH during the study period. Four hundred and ninety patients with at least one hepatitis B serology test were enrolled in this study, of which 265 were men and 225 were women. The median age was 52 years and ranged from 18 to 94 years. The median observation period was 596 days (ranged from one to 3065 days). The cytogenetic of this population compromised 234 patients with normal karyotype, 27 patients with t(8;21), 35 patients with t(15;17), 21 patients with inv(16), 13 patients with chromosomal 11q23 changes, 15 patients with del(5q)/monosomy5 or del(7q)/monosomy7, 57 patients with complex (i.e. more than three) chromosomal changes, and 80 patients with other simple chromosomal changes. There were 6 patients whose cytogenetic study revealed no mitosis and 2 patients without cytogenetic analysis. There were 23 patients with preceding history of myelodysplastic syndrome (MDS), 3 patients with therapy-related leukemia. There were 35 patients diagnosed as acute promyelocytic leukemia. The factors of cytogenetic change, leukemic subtype, preceding myelodysplastic syndrome and therapy-related were not significantly correlated with HBV reactivation and carrier status.

**Fig 1:**
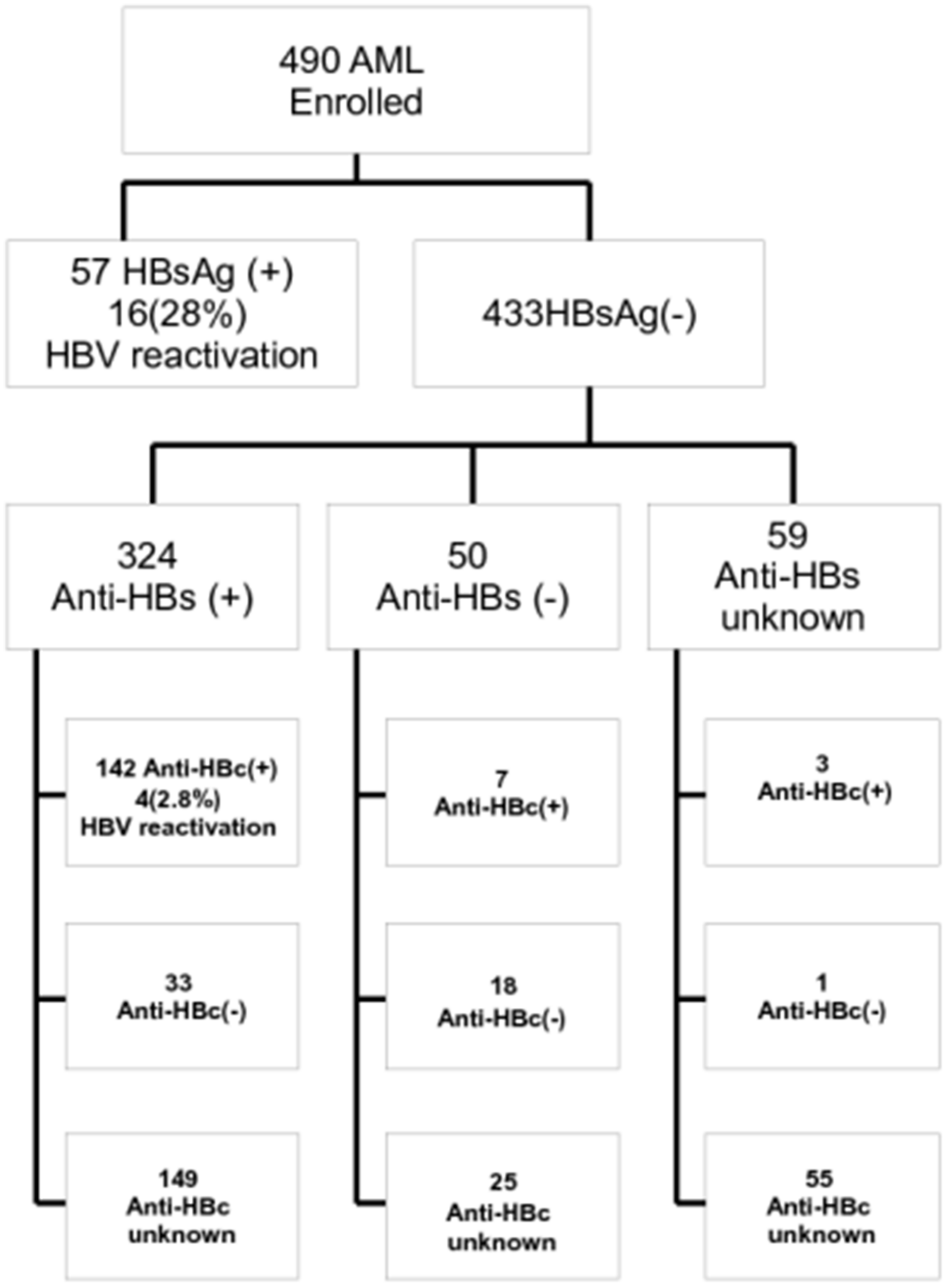
Study flow and hepatitis B serological data of 490 AML patients at Leukemia Diagnosis.

The clinical characteristics of these 490 AML patients are shown in Table 1. At the time of leukemia diagnosis, there were 57 patients with positive HBsAg giving an estimated prevalence of 11.6% and 433 patients with negative HBsAg (Fig 1). The anti-HBsAb serology was positive in 324 of 433 patients, negative in 50 patients, and not tested in 59 patients. Fifteen (3%) patients had chronic hepatitis C at diagnosis of leukemia and two patients had both hepatitis B and C. The incidence of hepatitis B reactivation and HBV-related hepatitis were 9.5 and 8.3 per 100 person-years among chronic HBV carriers of AML patients. Three of 32 HBV vaccinees who were born after July 1984 were HBV carriers.

**Table 1:** Clinical characteristics of 490 acute myeloid leukemia patients with positive and negative hepatitis B surface antigen (HBsAg).

	Positive HBsAg (n = 57)	Negative HBsAg (n = 433)	p value
Age			0.104
≧65 years	9	115	
<65 years	48	318	
Gender			0.482
Men	30	235	
Women	27	198	
Hypertension			0.593
Yes	9	86	
No	48	347	
Diabetes mellitus			0.492
Yes	4	46	
No	53	387	
HSCT*			0.205
Yes	20	114	
No	37	319	
Liver Cirrhosis			0.013
Yes	2	0	
No	55	433	
Hepatocellular carcinoma			0.013
Yes	2	0	
No	55	433	
HBV Vaccination			1.000
Yes	3	29	
No	54	404	
HBV reactivation			<0.001
Yes	16	4	
No	41	429	
Hepatitis C			0.690
Yes	2	13	
No	55	420	
Preceding MDS**			1.000
Yes	2	21	
No	55	412	
Acute promyelocytic leukemia			1.000
Yes	4	31	
No	53	402	
Cytogenetics***			0.210
Good	11	72	
Intermediate	30	284	
Poor	15	70	
unknown	1	7	
Initial Treatment			0.417
Supportive Care	2	24	
Standard dose chemotherapy	1	21	
Low dose chemotherapy	1	9	
Oral chemotherapy	48	313	
Hypomethylating agent	5	66	
Survival			0.393
Death	31	261	
Alive	26	172	

*HSCT: Hematopoietic stem cell transplantation;

**MDS: Myelodysplastic syndrome

***Cytogenetics: Good risk includes t(8;21), t(15;17), inv(16); high risk includes complex chromosomal changes(more than 3), del(5) / monosomy 5, del (7) /monosomy7, 11q23; Intermediate risk includes normal karyotype and others simple chromosomal change; unknown: 6 no mitosis, 2 not done.

### Clinical characteristics and outcome

Comparing HBs carriers to non-HBs patients, the median HBsAg was ≧ 250 IU/mL (n = 57, range 0.72 to ≧250 IU/mL) v.s 0 IU/mL (n = 433, range 0 to 0.04 IU/mL); median anti-HBsAb was 0.08 mIU/mL (n = 40, range 0 to 16.37 mIU/mL) v.s 90.21 mIU/mL (n = 374, range 0 to ≧1000 mIU/mL); median Anti-HBcAb was 12.75 (n = 17, range 10.58 to 18.12) v.s 3.97 (n = 204, range 0.17 to 20.74); median HBeAg was 0.32 (n = 33, range 0.16 to 1568.99) v.s 0.27 (n = 27, range 0.0 to 0.601); and median anti-HBeAb was 0.02 (n = 25, range 0.0 to 59.53) v.s 1.14 (n = 15, range 0.08 to 2.22), at initial diagnosis of leukemia, respectively. Twenty AML patients suffered from hepatitis B reactivation, including 16 (28%) of 57 patients with positive HBsAg and 4 (2.8%) of 142 patients with positive anti-HBsAb and anti-HBcAb. The median time to hepatitis B reactivation from time of leukemia diagnosis was 276 days (range from 32 to 718 days). Hepatitis B reactivation is significantly higher in AML patients with positive HBsAg than the patients with positive anti-HBsAb and anti-HBcAb (16/57 (28%), vs 4/142 (2.8%), p<0.001). The median HBV DNA viral load at the time of HBV reactivation was 1.88X10^6^ IU/mL. The baseline serum HBV DNA and HBsAg levels were not associated with HBV reactivation in HBsAg-positive patients. The median peak ALT level was 361 U/L (range from 51 to 2182) in the patients with hepatitis B reactivation. There were 18 patients with documented HBV-related hepatitis, including 14 patients who were chronic hepatitis B carriers and four patients with positive anti-HBsAb and anti-HBcAb. One HBsAg positive patients died of fulminant hepatitis B.

### Prophylaxis of Hepatitis B reactivation in the AML patients

Currently, there is no consensus for antiviral prophylaxis for AML patients with HBV carrier status in Taiwan. In clinical practice, antiviral prophylaxis generally starts at diagnosis of leukemia, and continues for at least one year after chemotherapy according to the clinical condition. Accordingly, forty-six of 57 positive HBsAg patients received HBV prophylaxis with antiviral agents after diagnosis of leukemia; 28 patients received lamivudine, 13 patients received entecavir, two patients received adefovir, two patients received telbivudine, and one patient received tenofovir. Ten patients were not prescribed any prophylaxis after diagnosis of leukemia. Overall, sixteen of 57 positive HBsAg patients suffered from recrudescence of hepatitis B virus during or after chemotherapy, including four patients who were antiviral-naive, seven patients who had discontinued initial HBV prophylaxis after a median of 118 days (range, 30 to 447 days), and five patients who were taking antiviral prophylaxis but still had hepatitis B reactivation. The latter five were under antiviral (lamivudine n = 2, adefovir n = 1, telbivudine n = 1, entecavir n = 1,) prophylaxis at the time of HBV reactivation, documented by a 10-fold or more elevation of hepatitis B viral load and accompanied by a median rise of ALT to 116 U/L, range 84–378 U/L). These five patients had good antiviral compliance. The durations from antiviral therapy to hepatitis B reactivation were 4, 12, 7, 14, and 3 months, respectively. Case 2 and case 4 had received hematopoietic stem cell transplantation (HSCT) and hepatitis B reactivation developed after 2 and 7 months after HSCT. The two patients under lamivudine prophylaxis with HBV reactivation were subsequently shown to harbor HBV virus with the YMDD mutation. Nevertheless, AML patients with positive HBsAg under antiviral prophylaxis had significantly decreased risks of hepatitis B reactivation than AML patients with positive HBsAg without prophylaxis (5 of 39 (13%), vs. 11 of 18 (61%), p<0.001).

### Risk Factors For hepatitis B reactivation

We compared the 20 patients who had hepatitis B reactivation and the other 419 patients who did not have hepatitis B reactivation to evaluate the risk factors for hepatitis B reactivation among patients previously exposed to HBV (Table 2). Fifty-one patients with negative anti-HBcAb were excluded. By univariate analysis, diabetes mellitus (p = 0.006), hematopoietic stem cell transplant (p = 0.014), liver cirrhosis (p = 0.002), positive HBsAg (p<0.001) and age less than 65 years (p = 0.020) appeared to be associated with HBV reactivation. By multivariate analysis, diabetes mellitus (p = 0.008, odds ratio (OR) = 2.841, 95% confident interval (CI): 0.985–8.193), and positive HBsAg (p<0.001, OR = 36.878, 95% CI: 11.770–115.547) were independent predictors of hepatitis B reactivation among AML patients.

**Table 2:** Overall risk factors for hepatitis B reactivation in 439 acute myeloid leukemia patients*.

	No Reactivation (n = 419)	Reactivation (n = 20)	Univariate P value	Multivariate P value	Odds ratio (95% CI)
Age			0.020	0.996	NA
<65 years	299	19			
≧65 years	120	1			
Gender			0.255		
Women	192	12			
Men	227	8			
Hypertension			0.778		
No	331	17			
Yes	88	3			
Diabetes			0.060	0.008	2.841 (0.985–8.193)
No	375	15			
Yes	44	5			
HSCT^a^			0.014	0.178	NA
No	321	10			
Yes	88	10			
Liver cirrhosis			0.002	>0.999	NA
No	419	18			
Yes	0	2			
Hepatocellular carcinoma			0.089	>0.999	NA
No	418	19			
Yes	1	1			
HBV Vaccination			>0.999		
No	398	19			
Yes	21	1			
Hepatitis B carrier			<0.001	<0.001	36.878 (11.770–115.547)
No	378	4			
Yes	41	16			
Hepatitis C			0.509		
No	405	19			
Yes	14	1			
Preceding MDS^b^			0.614		
No	398	20			
Yes	21	0			
APL^C^			>0.999		
No	387	19			
Yes	32	1			
Cytogenetic^d^			0.235		
Good	66	6			
Intermediate	270	10			
Poor	76	13			
Unknown	7	1			
Treatment			0.200		
Supportive	26	0			
Standard chemotherapy	293	19			
Low dose chemotherapy	68	1			
Oral chemotherapy	10	0			
Hypomethylating agent	22	0			
Survival			0.310		
Death	259	12			
Alive	160	8			

Abbreviation:

^a^HSCT: Hematopoietic stem cell transplantation;

^b^MDS (myelodysplastic syndrome);

^c^APL (Acute promyelocytic leukemia); NA(not available).

^d^Cytogenetic: Cytogenetics: Good risk includes t(8;21), t(15;17), inv(16); high risk includes complex chromosomal changes (more than 3), del(5) / monosomy 5, del (7) /monosomy7, 11q23; Intermediate risk includes normal karyotype and others simple chromosomal change; unknown: 6 no mitosis, 2 not done.

*51 patients with Anti-HBc(-) were excluded.

The subgroup analysis for HBV reactivation in 57 HBsAg-positive patients is shown in Table 3. Univariate analysis showed diabetes mellitus was associated with higher risk of HBV reactivation (p = 0.005) and antiviral therapy with less risk of hepatitis B reactivation (p<0.001). Multivariate analysis showed that HBsAg positive patients who had received antiviral therapy had significantly lower risk of hepatitis B reactivation (p = 0.033, odds ratio (OR) = 0.094, 95% confident interval (CI): 0.025–0.355).

**Table 3:** Risk factors for hepatitis B reactivation in the subgroup of AML patients with positive HBsAg (n = 57).

	Reactivation (n = 16)	No reactivation (n = 41)	Univariate P value	Multivariate P value	Odds ratio (95% CI)
Age			0.420		
≧65 years	1	8			
<65 years	15	33			
Gender			0.075	0.106	NA
Men	5	25			
Women	11	16			
Hypertension			1.000		
Yes	2	7			
No	14	34			
Diabetes mellitus			0.005	0.999	NA
Yes	4	0			
No	12	41			
HSCT			0.216		
Yes	8	12			
No	8	29			
Liver cirrhosis			0.075	0.999	NA
Yes	2	0			
No	14	41			
Hepatocellular carcinoma			0.486		
Yes	1	1			
No	15	40			
Antiviral prophylaxis			<0.001	0.033	0.094(0.025–0.355)
Yes	5	34			
No	11	7			
Hepatitis C			0.496		
Yes	1	1			
No	15	40			
Survival			1.000		
Death	9	22			
Alive	7	31			

HSCT: Hematopoietic stem cell transplantation, NA: not available

Four of 142 negative HBsAg AML patients had hepatitis B reactivation. Their unusual clinical courses are shown in Table 4. All four patients had seroconverted with positive anti-HBsAb and anti-HBcAb at the time of diagnosis of leukemia. Three of the four patients had relative lower anti-HBsAb serology titer before hepatitis B reactivation (24.95 IU/mL, 18.68 mIU / mL and 28.19 mIU / mL, respectively). Two patients (case no. 2 and no. 3) had received hematopoietic stem cell transplantation with long-term immunosuppressant medication and hepatitis B reactivation after 12 and 10 months of transplantation. Of the other two patients (case no. 1 and no. 4) who did not received transplantation, reactivation of hepatitis B occurred 7 and 11 months after chemotherapy. All four cases had hepatitis (the liver transaminases were greater than 100 U/L), and three died of leukemia within 4 months.

**Table 4:** The clinical course of four hepatitis B surface antigen negative AML patients who experienced hepatitis B reactivations despite lack of evidence of chronic HBV carriage.

Case	Age/ Gender Leukemiasubtype	HBsAg/anti-HBsAb (IU/mL / mIU/mL) at diagnosis of leukemia	Time Interval (month)	HBsAg/anti-HBsAb (IU/mL / mIU/mL) Follow-up	Time Interval (month)	HBsAg/anti-HBsAb (IU/mL / mIU/mL) Follow-up	Time Interval	Outcome
1	44/man AML, M6	0.03 / 24.95	7	14.67 / 0.41, (Post chemotherapy) HBV reactivation) HBVDNA: 7.95X10^4^IU/mL Anti-HBcAb: 11.84, HBeAg: 0.301, Anti-HBeAb: 0.02			1	Died of leukemia
2	30 / woman AML, M2	<0.05 / NA	5	NA / NA, (HSCT)	12	180.64 / NA, HBV reactivation, Anti-HBeAb: 25.92	84	Alive and leukemia free
3	55 / man AML, M1	0 / 1.64, Anti-HBcAb: 4.24	7	0 / 18.68, (2 months before HSCT), Anti-HBcAb: 5.43	12	> 250 / 0, HBV reactivation, Anti-HBcAb:3.75	1	Died of leukemia
4	46 / man AML, M5	0 / 194.16, HBeAg: 0.25	6	0 / 28.19, (Post chemotherapy), Anti-HBcAb:0.7	5	97.53 / 1.45, HBV reactivation, HBVDNA: 3.95X10^4^IU/mL, HBeAg: 443.15, Anti-HBeAb: 19.52	4	Died of leukemia

NA: not available

HSCT: hematopoietic stem cell transplantation

## Discussion

There is still controversy regarding the role of screening for hepatitis B reactivation in cancer patients. The American Society of Clinical Oncology Provisional Clinical Opinion reported that the evidence is insufficient to determine the net benefits and harms of routine screening for chronic HBV infection in individuals with cancer who are about to receive cytotoxic or immunosuppressive therapy or who are already receiving such therapy [22]. To the best of our knowledge, this is the first large cohort study to investigate hepatitis B reactivation in AML patients. In this study that was conducted in a country of HBV hyperendemicity, we showed that AML patients had a similar risk for HBsAg sero-positivity (11.6%), because most exposure to HBV that results in either development of anti-HBs antibody or chronic carriers of HBV in Taiwan before the implementation of the nationwide HBV vaccination program, occurred during the perinatal period or in early childhood. Our results showed that AML patients, especially those with diabetes and with HBsAg carriage, are also at high risk for hepatitis B reactivation. Prophylaxis with antiviral therapy significantly decreased the risk of hepatitis B reactivation. However, development of lamivudine resistance during antiviral therapy should be suspected if one observes a rebound of HBV plasma viral loads.

The incidence of hepatitis B reactivation and HBV-related hepatitis in our study were 9.5 and 8.3 per 100 person-years in AML patients who are also chronic hepatitis B carriers, which is similar to the incidence of hepatitis B reactivation in lymphoma patients (10.4 per 100 person-years) [11, 23]. While Nakamura *et al*. [24] reported that 10 of 13 (76.9%) AML patients had severe hepatitis due to HBV in 85 patients with hematological malignancy; Yeo *et al*. [10] found that patients with either leukemia or myeloma were at comparatively less risk of hepatitis B reactivation than patients with lymphoma. Since fulminant hepatitis B is a catastrophic event for HBsAg positive AML patients [6, 25, 26], periodic assessment of liver function and follow up of HBV serological status is important during chemotherapy. Further prospective cohort studies of patients with AML especially in the endemic hepatitis B area should be undertaken to capture the true incidence of HBV flare-ups.

In this study, AML patients with positive HBsAg under antiviral agents were significantly protected from hepatitis B reactivation. We therefore recommend that all HBsAg positive AML patients should be initiated on antiviral prophylaxis prior to chemotherapy. Although, there is no current consensus for antiviral prophylaxis for AML patients with HBV carrier status in Taiwan, the National Comprehensive Cancer Network (NCCN) guidelines indeed have some recommendation with regards to patients with high risk for HBV [27]. Moreover, the major HBV treatment guidelines proposals originating from America, Europe, and the Asia-Pacific region, recommended that all cancer patients should check HBV markers, including HBsAg and anti-HBc, prior to initiation of chemotherapy, and to use routine prophylactic antiviral therapy for individuals who are positive for HBsAg before the start of cancer chemotherapy [28–30]. The optimal timing, duration and choice of anti-HBV agent for prophylaxis remain unknown [30–32]. In our study cohort, despite antiviral prophylaxis, seven AML patients experienced hepatitis B reactivation after discontinuation of prophylaxis. Hence, close monitoring of the liver functional assays as well as hepatitis B plasma viral loads may be indicated in the first year after discontinuation of antiviral prophylaxis in patients with ongoing immune suppression. Of the patients with reactivation under ongoing anti-HBV prophylaxis, two of the four patients underwent genotypic resistance testing. Both patients had proven YMDD (rtM204I/V) mutation under lamivudine prophylaxis. Hence, the risk for emerging resistance should be considered in the event of virologic breakthrough during lamivudine therapy. Resistance to other anti-HBV viral agent in the first year is less frequently encountered.

In this study, four patients with positive anti-HBsAb lost seroprotection with reappearance of HBsAg. Of cancer patients receiving chemotherapy or transplantation, anti-HBsAb levels might decline below the threshold for protection against new HBV infection or reactivation [33–36]. Although HBV vaccination is highly immunogenic and efficacious in inducing protective antibody against HBV [37], a gradual decline in titers decades after vaccination have been reported [38]. Moreover, serologic evidence of recovery from hepatitis B infection does not preclude its reactivation after immunosuppression. Thus, screening for serologic evidence of hepatitis B and booster HBV vaccination for those without protective anti-HBsAb should be considered in individuals in whom immunosuppressive therapy is planned.

The finding that diabetes mellitus is associated with HBV reactivation is biologically plausible since the hyperglycemic environment increases the virulence of viral pathogens. The underlying mechanisms may involve reduced production of interleukins in response to infection, reduced chemotaxis and phagocytic activity, and reduced humoral immunity [39]. Wang et al. reported diabetes was associated with serum alanine aminotransferase activity elevation in patients with hepatitis B infection [40]. Diabetes mellitus has also been documented as an independent risk factor for HBV related hepatocellular carcinoma [41] and occult HBV infection [42]. In general, diabetic patients are considered to be relatively immunocompromised. However the exact role of hyperglycemia in hepatitis B reactivation should be further clarified.

This study suffered from several limitations. Since the study is a retrospective review, the clinical data was derived from medical records in accordance to clinical practice. Therefore, data were limited or unavailable for several variables. Hence, subclinical hepatitis or asymptomatic reactivations, newly acquired HBV infection or seroconversion of HBsAg may be potentially missed. In addition, patients with early mortality during AML treatment may have contributed to underestimated risks of HBV reactivation.

In conclusion, hepatitis B virus on reactivation is not uncommon in HBsAg positive AML patients receiving or undergoing chemotherapy. Prophylaxis or early preemption with an anti-HBV agent significantly reduced the risk of hepatitis B reactivation and HBV related hepatitis. Diabetes mellitus and chronic hepatitis B carrier status were independent risk factors of hepatitis B reactivation in AML patients.
